# Minimally Invasive Treatment of a Cervical Aneurysmal Bone Cyst Through Percutaneous Doxycycline-Albumin Foam Injection

**DOI:** 10.1007/s00270-022-03292-y

**Published:** 2022-11-01

**Authors:** Fernando Bueno Neves, Olav Jansen, Jens Trentmann, Karim Mostafa, Jawid Madjidyar

**Affiliations:** 1 Klinik Für Radiologie und Neuroradiologie, Universitätsklinikum Schleswig-Holstein, Arnold-Heller-Straße 3, 24103 Kiel, Germany; 2 Klinik Für Neuroradiologie, Universitätsspital Zürich, Frauenklinikstrasse 10, 8091 Zurich, Switzerland

To the Editor,

We report the case of a 28-year-old male with no medical history presenting with cervical pain over a month. Initial MRI and CT findings showed a lobulated and cystic lesion of the fourth cervical vertebra with thin cortical walls, around 2,2 × 3,5 × 4,2 cm, with blood-fluid levels consistent with an aneurysmal bone cyst (ABC) (Fig. 1). The vertebra lacked substantial bone tissue and was considered fracture prone. Multi-centric evaluation revealed a high-risk operation since the right vertebral artery was encased in the lesion and would have to be sacrificed. Transarterial embolization was considered too risky due to the situation of the anterior spinal artery (Fig. 2) and surgical stabilization would be a risky and only palliative option. Percutaneous doxycycline therapy was then considered the best curative option in this case.

**Fig. 1:**
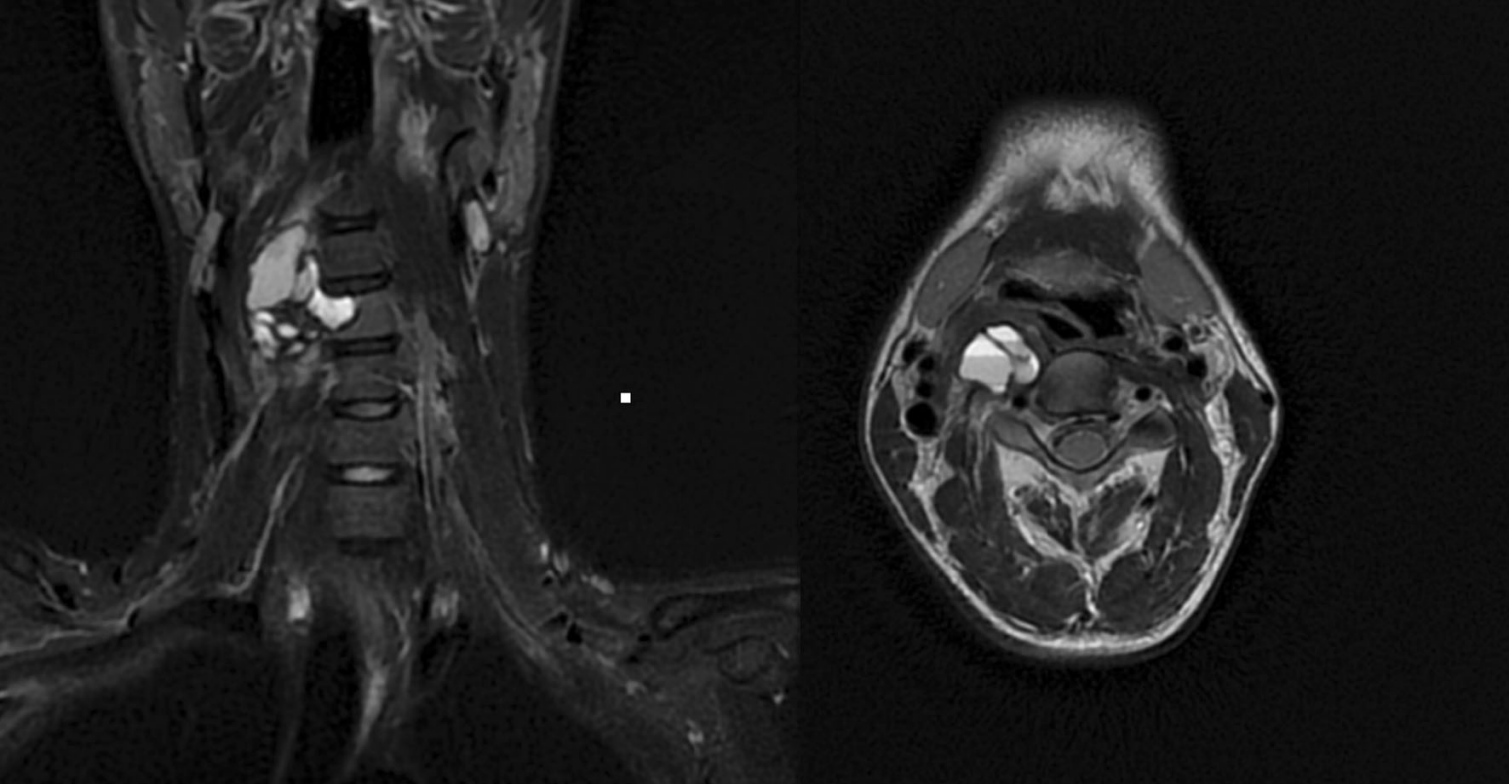
Pre-interventional MRI depicting an aneurysmal bone cyst of the fourth cervical vertebra, around 2,2 × 3,5 × 4,2 cm, with characteristic blood-fluid levels

**Fig. 2:**
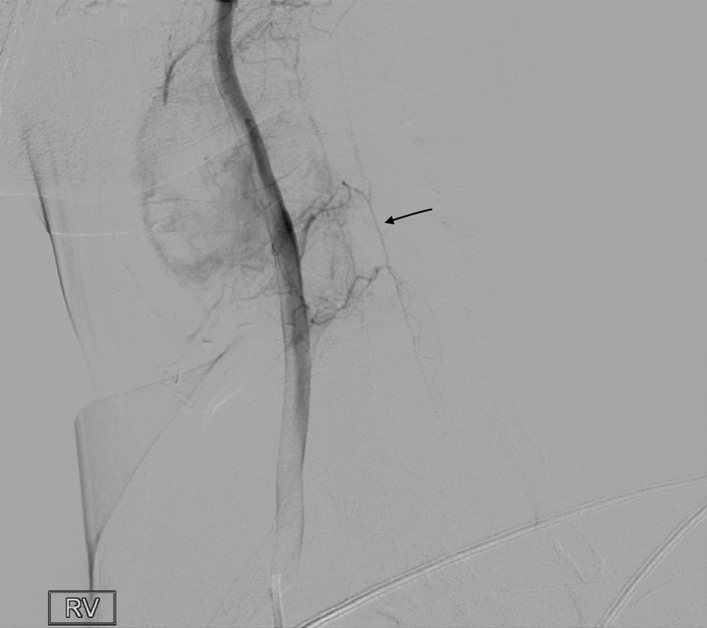
Diagnostic angiography depicting the right vertebral artery and its multiple ectatic branches that supply part of the aneurysmal bone cyst (ABC) walls. The anterior spinal artery (black arrow) is also visible next to the ABC (colour figure online)

The intervention was performed under conebeam CT (CBCT) and fluoroscopy guidance (Siemens Artis zee), under general anesthesia and in a prone position. Two 18G needles were placed into each cystic chamber and digital subtraction angiography confirmed their correct position. Doxycycline (20 mg/ml) and human albumin (20%) were mixed 1:1 with air in equal proportions to produce doxycycline foam, which was then applied via 3 ml luer-lock syringes into each cyst (total 7 ml). While the foam was injected via one needle, the other served as a drain to avoid overpressure. Post-interventional CBCT depicted a foam distribution in 50% of the tumor volume with minor reflux into the epidural and periradicular venous plexus. A second session was carried out five months later targeting the untreated parts of the ABC. One year after the procedure, CT imaging depicted almost complete ossification of the ABC with minor cystic remnants (Fig. 3). The facet joints of C3-C5 showed an asymptomatic fusion. No symptoms were observed in the follow-up.

**Fig. 3:**
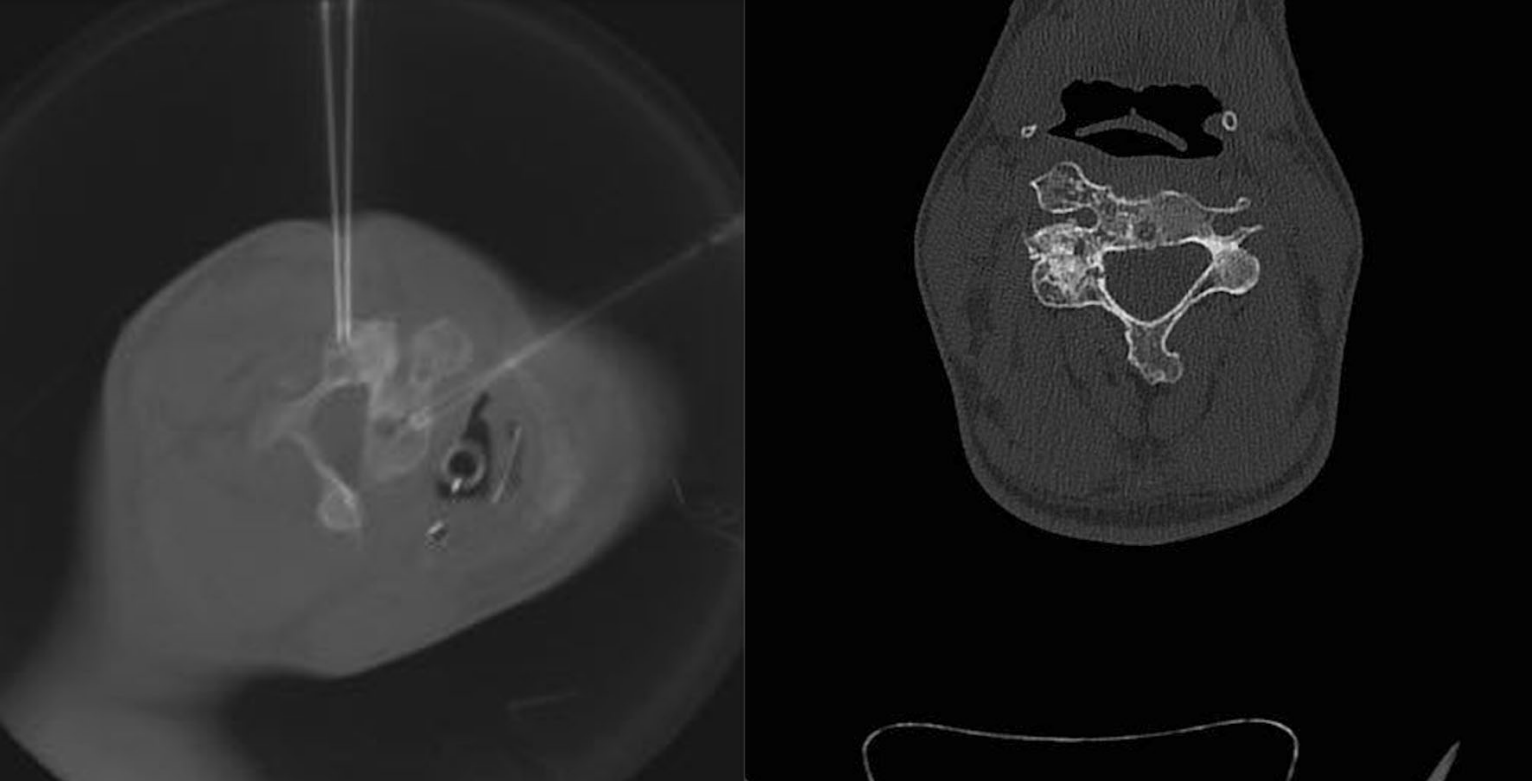
Needle placement inside the cystic chambers during the second sclerotherapy session (left) and CT image one year after the procedure depicting almost complete sclerosis within the cystic chambers (right)

ABCs are rare entities comprising blood-filled spaces separated by connective or osteoid tissue with high activity of giant osteoclast cells. A diagnostic biopsy is mandatory to define whether the ABC is a primary lesion—which was the case, since a biopsy was performed prior to the intervention—or secondary to underlying conditions such as osteosarcomas and chondroblastomas. The cysts are directly connected to the venous system, which could be illustrated in this case by the reflux into the epidural and periradicular venous plexus. The broad therapeutic spectrum includes en bloc resection and stabilization, radiotherapy, coagulation, cryosurgery, and selective arterial embolization [1, 2]. Sclerotherapy seems to be a viable alternative to high-risk operations, with reported recurrence rates of around 5% at more than 24 months [3]. Toxicity does not arise from doxycycline in the described concentration. Over-pressurizing might cause reflux into the vertebral artery and eventually into the anterior spinal artery, which may lead to paralysis. Sequential treatment sessions may be necessary if the lesion cannot be treated completely during the first session.

Percutaneous doxycycline foam therapy might be a viable option for complex vertebral ABCs. The major limitation is the lack of robust data, since the current evidence level derives mainly from case reports and case series.

## References

[1] Park HY, Yang SK, Sheppard WL (2016). Current management of aneurysmal bone cysts. Curr Rev Musculoskelet Med.

[2] Rossi G, Rimondi E, Bartalena T, Gerardi A, Alberghini M, Staals EL, Errani C, Bianchi G, Toscano A, Mercuri M, Vanel D (2010). Selective arterial embolization of 36 aneurysmal bone cysts of the skeleton with N-2-butyl cyanoacrylate. Skeletal Radiol.

[3] Shiels WE, Mayerson JL (2013). Percutaneous doxycycline treatment of aneurysmal bone cysts with low recurrence rate: a preliminary report. Clin Orthop Relat Res.

